# Cholera outbreak and water resources: Linkages between consumption, infection spread at Mehar village, Sagar district in India

**DOI:** 10.6026/973206300221834

**Published:** 2026-03-31

**Authors:** Shailendra Kumar Patne, Durgesh Kumar Sharma, Indu Jyotsna Ekka, Kavita N Singh

**Affiliations:** 1 Department of Community Medicine, Gandhi Medical College, Bhopal, Madhya Pradesh, India; 2 Department of Obstetrics and Gynaecology, Gandhi Medical College, Bhopal, Madhya Pradesh, India

**Keywords:** Cholera outbreak, *Vibrio cholerae*, water resources, epidemic investigation

## Abstract

A cholera outbreak in Mehar village, Sagar District, Madhya Pradesh (July 2024) affected 901 individuals (attack rate 18.02%) with 4 deaths among elderly comorbid patients. Epidemic investigation confirmed *Vibrio cholerae* and *faecal coliforms* in rectal swabs, stool samples and water from the community bore well serving as the primary drinking source near the temple. Despite multiple alternative water sources available, this single bore well supplied most households, schoolchildren (37%) and open defecation- practising communities (80%). Contaminated bore well water represented the point source of outbreak, exacerbated by open defecation despite absence of visible contamination markers nearby. Thus, we show outbreak investigation methodology by demonstrating integrated water source tracing and rapid microbiological confirmation as essential strategies for cholera containment in rural settings.

## Background:

Epidemics of acute vomiting and diarrhoea with mild to severe dehydration occur frequently during rainy seasons in developing countries, including India [1]. Cholera, caused by the bacterium *Vibrio cholerae* of serogroups O1 or O139, is an acute diarrhoeal infection that spreads through contaminated food and water, capable of causing death within hours if left untreated [2]. Seven cholera pandemics have occurred since 1817, with the seventh pandemic originating in Indonesia in 1961 and subsequently spreading across Asia, the Middle East, Africa and the Americas [3]. The World Health Organisation reported 535,321 cases and 4,007 deaths from 45 countries in 2023, indicating cholera remains a significant global public health threat [4].The Indian subcontinent is particularly vulnerable to cholera due to its vast coastlines, areas with poor sanitation, unsafe drinking water and overcrowding [5]. Between 2011 and 2020, India reported 565 cholera outbreaks affecting over 45,000 cases, with 90% occurring in rural areas lacking proper sanitation and health facilities [6]. Cholera outbreaks are strongly associated with inadequate water, sanitation and hygiene (WASH) services, with risk factors including drinking from unprotected water sources, limited hygiene facilities, lack of soap and water and open defecation [7]. Although India's Swachh Bharat Mission, initiated in 2014, aimed to provide rural and urban poor communities with toilet subsidies, not all communities have benefited from this scheme [8]. *Vibrio cholerae* inhabits aquatic environments and can be transmitted not only through contaminated water but also through consumption of raw or undercooked seafood, particularly shellfish and fish that harbour the pathogen [9]. Therefore, it is of interest to describe this cholera outbreak investigation in Mehar village, Sagar District, Madhya Pradesh, to elucidate the linkages between water resources, fish consumption and infection spread among vulnerable rural communities.

## Materials and Methods:

## Study setting and outbreak notification:

A diarrhoea outbreak was reported in Mehar village, Rahatgarh tehsil, located 35 km from Sagar city, Madhya Pradesh, India, in July 2024 [10]. The village population was approximately 4,500 individuals residing in 750 households across six community habitations: Andar Basti, Raikwar Mohalla, Road Mohalla, Harijan Mohalla, Ram Mohalla and Naranpura Mohalla. Following public protests and news of four deaths, the State surveillance team conducted an epidemic investigation on the 4th day of the outbreak (9 July 2024).

## Epidemic investigation:

The investigation team visited multiple sites, including the Health and Wellness Centre of Mehar village, the village health base camp established at a local school, District Hospital Sagar and Bundelkhand Medical College. House-to-house surveys were conducted to identify cases and assess sanitary conditions.

## Case definition:

A case was defined as any resident of Mehar village presenting with three or more episodes of acute watery diarrhoea with or without vomiting within 24 hours between 2-10 July 2024.

## Data collection:

Secondary data were reviewed from cases registers maintained at the health base camp, Health and Wellness Centre, District Hospital and Medical College. Information collected included demographic details, date of onset, clinical presentation and household address.

## Laboratory investigation:

Rectal swab samples were collected from symptomatic patients for culture and sensitivity testing. Water samples were collected from the suspected community borewell, private borewells and open wells for bacteriological analysis to identify *Vibrio cholerae* and *faecal coliforms* bacteria.

## Sanitary survey:

A rapid sanitary assessment was conducted by the Public Health Engineering team and the Chief Executive Officer of the village panchayat to evaluate water sources, toilet functionality and hygiene practices among households.

## Statistical analysis:

Attack rates were calculated as the number of cases per population at risk. Case distribution was analysed by age, sex and residential location. Data were analysed using descriptive statistics.

## Results:

The outbreak was confirmed through reports to the District Hospital, base camp and Health and Wellness Centre Mehar. The first reported case was a 6-year-old female admitted to Bundelkhand Medical College on 3rd July 2024, who presented with a sudden onset of acute nausea, vomiting and severe dehydration within 2 hours of consuming water from the suspected community borewell. A total of 901 cases were reported between 2nd and 10th July 2024, with peak incidence on 4th and 5th July (160 and 335 cases respectively), indicating rapid transmission within the incubation period of 2 hours to 2 days. The attack rate was 18.02 per 1000 population, with a high secondary attack rate observed after exposure to primary cases (Table 1, Figure 1 - see PDF). Age and sex distribution analysis of 657 cases revealed that school-going children aged 6-15 years were most affected (37%), followed by other age groups, with both sexes equally susceptible (Table 2, Figure 2 - see PDF). Place-wise distribution showed Harijan Mohalla (38.46%) andar Basti (31.87%) and Narayanpura (25.27%) as the most affected areas, while Raikwar Mohalla and Road Mohalla accounted for only 4.4% of cases (Table 3, Figure 3 - see PDF). The suspected community bore pump near the temple wall was the primary water source for more than 70% of the village population and school children were dependent on water stored from this source due to non-functional borewells in the school premises (Table 4 - see PDF). Investigation revealed that a fisher family residing in Harijan Mohalla had consumed partially cooked fish from Ghassan River on 2nd July 2024 and subsequently developed diarrhoea and vomiting; this family had also sold fish to 8 families in Narayanpura Mohalla who similarly developed acute diarrhoeal symptoms (Table 5, 6 - see PDF). Four deaths occurred during the outbreak, all among elderly individuals (three females, one male) with low immune status and comorbid conditions (Table 7 - see PDF).

## Discussion:

This outbreak investigation confirms a point-source cholera epidemic in Mehar village with secondary spread through contaminated fish consumption and person-to-person transmission. The attack rate of 18.02 per 1000 population is consistent with cholera outbreaks reported in rural India, where inadequate water, sanitation and hygiene (WASH) infrastructure facilitates rapid disease transmission [10]. Similar attack rates ranging from 15-25 per 1000 have been documented in cholera outbreaks across South Asia, particularly in communities with limited access to safe drinking water [11]. The identification of *Vibrio cholerae* in stool samples and faecal coliform bacteria (*E. coli*) in water samples establishes the aetiology of this outbreak. The initial negative water sample from the community borewell suggests contamination occurred through an infected individual using the water source, rather than direct *faecalcoliform* contamination of the well [12]. This finding aligns with evidence that asymptomatic carriers, who constitute approximately 75% of infected individuals, can shed *Vibrio cholerae* in their *faeces* for 7-14 days and contaminate water sources during the acute phase of infection [13]. Studies have demonstrated that a single symptomatic cholera patient can excrete up to 10-20 litres of rice-water stool per day containing 10^7 to 10^9 *vibrios* per millilitre, making contamination of shared water sources highly efficient [14]. The epidemic curve showing peak incidence on 4th and 5th July 2024 (160 and 335 cases respectively) is characteristic of a point-source outbreak with subsequent propagated transmission. The rapid rise in cases within the typical incubation period of 2 hours to 5 days indicates a common source exposure followed by secondary person-to-person spread [15]. This biphasic pattern has been observed in several cholera outbreaks where initial contamination of a water source leads to primary cases, followed by household-level transmission through contaminated hands, food and fomites [16]. The disproportionate burden on school-going children aged 6-15 years (37%) reflects their dependence on the community borewell due to non-functional school water sources. Studies from India have reported similar age distributions in cholera outbreaks, with children being particularly vulnerable due to lower immunity, higher exposure to contaminated water at schools and inadequate hand hygiene practices [17]. A systematic review of cholera outbreaks in endemic regions found that school-aged children have 1.5-2 times higher risk of infection compared to adults, primarily due to behavioural factors and congregate settings [18]. The storage of water from the suspected borewell by school staff further amplified exposure among this vulnerable population. The role of contaminated fish in disease transmission represents a critical finding of this investigation. *Vibrio cholerae* is known to inhabit freshwater and brackish water environments, forming biofilms on aquatic organisms, including fish, shellfish, crustaceans and zooplankton [19]. The bacterium can survive and multiply in fish intestines and on fish surfaces, particularly when fish are harvested from waters contaminated with human faecal matter [20]. The temporal association between fish consumption on 2nd July and subsequent illness among the fisherman's family and customers in Narayanpura strongly supports fish-mediated transmission as a secondary amplification route. Similar outbreaks linked to consumption of raw or partially cooked fish have been documented in Bangladesh, Haiti and several African countries [21]. The concentration of cases in Harijan Mohalla (38.46%) and Narayanpura (25.27%) correlates strongly with poor sanitation practices, where 80% of households lacked functional toilets. Open defecation along the Ghassan River likely contributed to environmental contamination and subsequent fish infection, creating a transmission cycle linking sanitation failures to foodborne spread [22]. The practice of using river water and mud for hand washing after defecation, instead of soap, further perpetuates faecal-oral transmission. Studies have consistently demonstrated that open defecation increases cholera risk by 2-3 folds compared to communities with improved sanitation and hand washing with soap reduces diarrheal disease transmission by 42-47% [23]. The failure of the Swachh Bharat Mission to provide functional toilets in this community highlights implementation gaps in rural sanitation programs. Despite India's ambitious sanitation goals, studies have shown that toilet construction alone is insufficient without concurrent behaviour change communication, regular maintenance and water availability for toilet use [24]. In Mehar village, the non-functional status of constructed toilets due to water scarcity and poor maintenance forced households to revert to open defecation, negating the intended public health benefits. The non-functional status of the Nal Jal Yojana (piped water scheme) overhead tank and the dependence of over 70% of the population on a single community borewell created conditions ideal for outbreak propagation. Centralised water sources without adequate protection and chlorination have been implicated in numerous cholera outbreaks globally [25]. The World Health Organisation recommends household water treatment and safe storage as interim measures in communities lacking a reliable piped water supply, combined with point-of-use chlorination during outbreaks [26]. The four deaths among elderly patients with comorbidities highlight the importance of rapid case identification and referral protocols during outbreaks. Cholera case fatality rates can exceed 50% in untreated cases but should remain below 1% with prompt and adequate rehydration therapy [27]. Delays in initiating intravenous rehydration therapy, particularly in patients with severe dehydration and underlying conditions such as diabetes, cardiovascular disease and malnutrition, significantly increase mortality risk [28]. The establishment of oral rehydration corners at the village health base camp likely prevented additional deaths by providing early treatment access. This outbreak also underscores the importance of integrated disease surveillance and rapid response systems. The 4-day delay between the first case (3rd July) and the State surveillance team investigation (9th July) allowed substantial disease amplification. Studies have shown that early outbreak detection and response within 48-72 hours can reduce case numbers by 50-80% through targeted interventions including water source chlorination, case isolation and chemoprophylaxis of contacts [29]. Strengthening the Integrated Disease Surveillance Programme (IDSP) at the primary health centre level and ensuring timely reporting through the existing network is essential for future outbreak prevention. The socioeconomic dimensions of this outbreak deserve attention. The predominance of cases among low-caste communities engaged in fishing occupations reflects broader patterns of health inequity in India, where marginalised populations bear disproportionate burdens of preventable infectious diseases [30]. Addressing cholera and other waterborne diseases requires not only technical interventions but also social policies that ensure equitable access to safe water, sanitation and healthcare services across all community segments. This investigation relied on secondary data from health facility registers, which may underestimate true case numbers due to unreported mild cases and home-based treatment. Microbiological confirmation was limited to a few samples due to logistical constraints and delayed sample collection. The exact timing and mechanism of borewell contamination could not be definitively established. Additionally, detailed dietary histories and water consumption patterns were not systematically collected for all cases, limiting the precision of exposure assessment.

## Conclusion:

Cholera outbreaks can spread through contaminated fish even without visible water contamination, emphasising integrated environmental and food surveillance. Concentrated cases among marginalised groups highlight the need for sanitation programs coupled with behavioural change initiatives in rural India. Strengthening piped water supply, outbreak detection and referral protocols across all health facility levels is vital to prevent future epidemic mortality.
